# Global-Token U-Net with Hybrid Loss for Trustworthy Medical Image Super-Resolution

**DOI:** 10.3390/s26051454

**Published:** 2026-02-26

**Authors:** Jiaqi Shang, Zhiyuan Xu, Dongdong Wang

**Affiliations:** 1 Department of Mechanical Engineering, Hohai University, Nanjing 211100, China; 2 School of Mechanical Engineering, Nanjing University of Science and Technology, Nanjing 210094, China

**Keywords:** medical image processing, super-resolution, trustworthy AI, hybrid loss function

## Abstract

Super-resolution technology significantly enhances the visual quality of low-resolution medical images, resulting in ultra-high-resolution clear images. Super-resolution technology based on artificial intelligence has achieved great success in reconstruction quality. However, like the image restoration task, super-resolution is also an ill-posed problem, and current work lacks consideration of trustworthiness. Medical image super-resolution needs to ensure clarity and, more importantly, to ensure that the output image is reliable and does not produce false details and mislead the diagnosis. To address the trustworthy issue of medical image super-resolution, we design a novel hybrid loss that combines a hinge-based adversarial term with a PSNR-based regularization. In the designed loss function, the adversarial term makes the reconstructed result close to the distribution of the true high-resolution image, thus generating more refined high-frequency textures, while the PSNR-based regularization term explicitly reduces the deviation from the ground truth. We apply this loss in the global-token U-Net backbone network and add a lightweight VGG as the discriminator for adversarial terms. We empirically verify that integrating the proposed methods can enhance the trustworthiness of medical image super-resolution technology while maintaining high reconstruction quality.

## 1. Introduction

**Figure 1:**
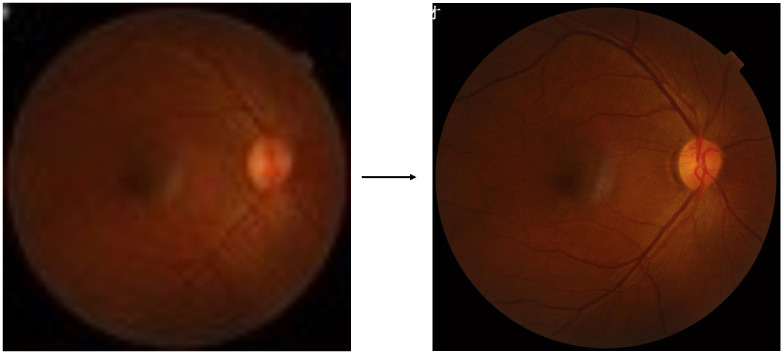
Medical image super-resolution example.

Super-resolution is an image processing technology aimed at significantly enhancing the resolution of images, thereby improving visual quality [1,2]. For medical imaging, low-resolution medical images can hinder diagnosis. Super-resolution technology can bring additional knowledge to improve the diagnostic effect [3]. As shown in Figure 1, super-resolution technology can significantly improve the visual quality of blurred medical images. Higher visual knowledge can assist human experts in diagnosis and can also be used to enhance the performance of advanced machine intelligence tasks, such as further instance segmentation and classification.

Dong et al. used convolutional neural networks to achieve universal image super-resolution [4], which was the first time that artificial intelligence technology was applied in the field of image super-resolution. Since then, super-resolution methods based on artificial intelligence have become mainstream, and many new models and methods have been proposed [5,6,7,8]. With the development of intelligent medical care, dedicated super-resolution models for medical images have also been proposed, achieving ideal results [9,10,11].

**Figure 2:**
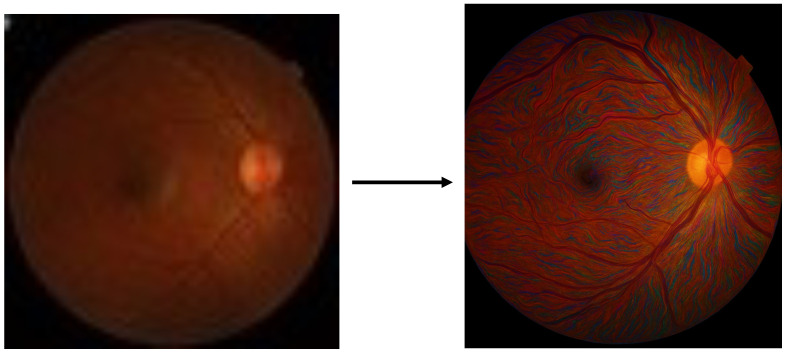
An extreme example of a super-resolution hallucination generated using our other visual synthesis models.

Deep learning has achieved great success in image restoration fields, including super-resolution [12,13]. However, all image restoration technologies based on artificial intelligence involve the process of deep scene synthesis. Scene synthesis models with high perceptual quality often generate results that are difficult to distinguish from real data; that is, details that seem clear but do not actually exist appear [14,15]. In the medical field, the consequences of this kind of image hallucination are serious [16]. Figure 2 is an extreme example of super-resolution using our other high-perception visual models. It can be seen that the super-resolution result looks very clear, but there are a lot of falsified details. This kind of medical imaging will hinder the diagnostic process and draw our attention to trustworthiness.

In this paper, we propose a method to enhance the trustworthiness of medical image super-resolution. Prior works have pointed out that medical images have global features [17,18]. Considering long-distance semantics, we adopt global-token U-Net as the backbone network. We design a new hybrid loss function that combines PSNR loss [19] and adversarial loss [20]. The adversarial term uses a lightweight VGG as the discriminator, mainly considering the reconstruction effect to generate fine and clear textures. The PSNR term uses explicit regularization constraints to reduce the difference between the output results and the ground truth. With this combination of hybrid loss and global modeling, we can achieve a sufficiently clear super-resolution result and improve the level of trustworthiness. The main contributions of this work are threefold:

- We revisit medical image super-resolution from the perspective of trustworthiness and identify hallucinated high-frequency details as a critical failure mode of deep learning-based restoration for ill-posed inverse problems. We argue that trustworthy medical image super-resolution should jointly pursue visual clarity and anatomical fidelity, rather than perceptual quality alone.
- We propose a global-token U-Net-based generator and a novel hybrid loss, which jointly aim to achieve trustworthy medical image super-resolution by capturing global anatomical context and suppressing hallucinated high-frequency details. The backbone augments a U-shaped convolutional network with global tokens to efficiently capture long-range anatomical context, while the hybrid loss combines a hinge-based adversarial term driven by a lightweight VGG discriminator with a PSNR-based regularization term to enhance perceptual sharpness and explicitly suppress hallucinated details.
- We conduct extensive experiments on medical image super-resolution benchmarks, showing that the proposed method maintains high reconstruction accuracy while achieving consistently perceptual quality. Qualitative and quantitative analyses further demonstrate that our approach produces fewer falsified structures and improves the trustworthiness of the reconstructed medical images.

The remainder of this paper is organized as follows: Section 2 reviews related work on universal and medical image super-resolution and discusses hallucination risks in ill-posed restoration. Section 3 presents the proposed global-token U-Net architecture and the hybrid adversarial–PSNR loss. Section 4 reports experimental settings and quantitative/qualitative results, including cross-domain evaluation and ablation studies. Finally, Section 5 concludes this paper and outlines future directions.

## 2. Related Work

### 2.1. Universal Image Super-Resolution

Early image super-resolution methods were mainly based on computer graphics and multimedia information processing technology [21]. Later, convolutional neural networks achieved outstanding performance in various computer vision tasks, and some super-resolution methods based on convolutional networks were proposed [4,5]. Modern super-resolution techniques mainly employ models with strong representational capabilities, such as transformers [8,22,23] and diffusion models [1,7].

The learning-based works include specific models for super-resolution and universal models for image restoration, and both achieve very clear reconstruction results. However, these works lack consideration of trustworthiness, which limits their application in real-world scenarios.

### 2.2. Medical Image Super-Resolution

Medical imaging has unique proprietary prior knowledge, so medical image super-resolution models can be designed [24,25,26,27]. Some common medical image restoration techniques also take into account super-resolution [11,18]. The acquisition of medical images involves multiple modalities and higher dimensions. Therefore, researchers have designed super-resolution models for multimodal medical imaging [10,28,29].

Hallucinations can lead to serious consequences, so the reconstruction of medical images has very strict requirements for trustworthiness [16]. This inspires us to propose novel solutions for trusted medical image super-resolution.

### 2.3. Ill-Posed Inverse Problems and Hallucination in Restoration

Image restoration tasks such as denoising, deblurring, inpainting, and super-resolution can all be formulated as inverse problems of an underlying degradation process. Image restoration models are often more concerned with synthetic datasets, and models that perform well in their settings may not be effective in real-world applications.

Learning-based image restoration is clearly an ill-posed problem. Restoring high visual quality from a degraded image may yield different results, and these seemingly high-quality clear images may contain details that do not exist in real scenes. Cohen et al. provide an information-theoretic analysis of hallucinations in generative restoration models, revealing that there is a certain contradiction between better perceptual quality and keeping true details [15].

In medical imaging, such hallucinated structures can have serious consequences. Bhadra et al. point out that images reconstructed by intelligent models can lead to false clear details that mislead the diagnosis [16]. As a deep synthesis reconstruction technology, super-resolution also faces similar risks. Therefore, it is important to design restoration schemes that explicitly account for the ill-posed nature of the problem and control hallucinated content. This perspective motivates us to focus on trustworthy medical image super-resolution and to develop methods that balance visual clarity with fidelity.

### 2.4. Discussion

**Super-resolution is an inherently ill-posed inverse problem: multiple high-resolution images can correspond to the same low-resolution observation under the degradation operator, making the mapping from LR to HR non-unique and under-constrained.** Based on the above analysis of hallucination, we discuss the hallucination risk for the methods in Section 2.1 and Section 2.2 and summarize the results in Table 1. The synthetic paths for generating processing results using different methods are different, which leads to different risks of false details and other credibility issues. This work aims to enhance the credibility of super-resolution by imposing constraints on the loss function.

## 3. Method

### 3.1. Model Architecture

**Figure 3:**
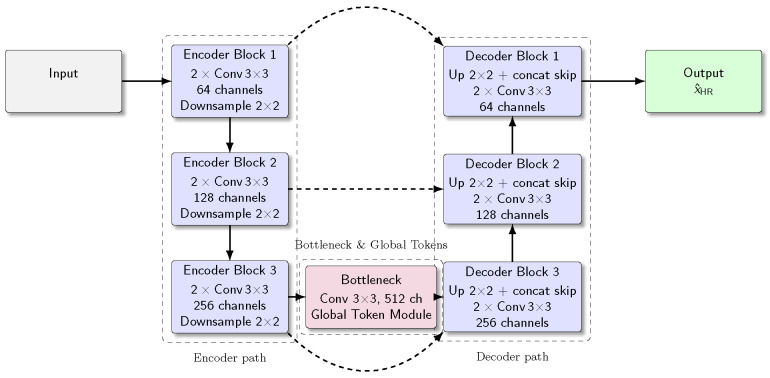
The architecture of global-token U-Net. At the bottleneck layer, we choose the number of attention heads to be 4 and the number of global token layers to be 4.

We adopt the classic U-Net [30] architecture and add global-token-aware mechanisms at the bottleneck layer as the backbone network. The pipeline is shown in Figure 3. At the bottleneck level, given the encoder feature $F_{S}\in R^{C\times H\times W}$, we first project it into a sequence of local tokens via a 3×3 convolution and flattening:

(1) $$X^{\left(0\right)}=\Phi \;\left(F_{S}\right)\in R^{N\times d},\;N=H\cdot W,$$

where Φ(·) denotes the projection and flattening operator, *d* is the token dimension, and each row of $X^{\left(0\right)}$ is a local token.

We introduce *K* learnable global tokens

(2) $$G^{\left(0\right)}\in R^{K\times d},$$

and form the initial token matrix by concatenation

(3) $$Z^{\left(0\right)}=\left[\begin{array}{c} G^{\left(0\right)}\\ X^{\left(0\right)} \end{array}\right]\in R^{(K+N)\times d}.$$

The global-token module applies *T* layers of multi-head self-attention to $Z^{\left(0\right)}$. For t=1,…,T, we write

(4) $$Z^{\left(t\right)}=Z^{\left(t-1\right)}+{\mathrm{MSA}}^{\left(t\right)}\;\left(Z^{\left(t-1\right)}\right),$$

where ${\mathrm{MSA}}^{\left(t\right)}\left(\cdot \right)$ denotes multi-head self-attention. Given an input token matrix $Z\in R^{(K+N)\times d}$, the multi-head self-attention is defined as

(5) $$\begin{array}{cc} {\mathrm{MSA}}^{\left(t\right)}\left(Z\right)\; & =\mathrm{Concat}\left(H_{1}^{\left(t\right)},\ldots ,H_{M}^{\left(t\right)}\right)\;W_{O}^{\left(t\right)}, \end{array}$$

(6) $$\begin{array}{cc} H_{m}^{\left(t\right)}\; & =\mathrm{Softmax}\;\left(\frac{Q_{m}^{\left(t\right)}{\left(K_{m}^{\left(t\right)}\right)}^{⊤}}{\sqrt{d_{m}}}\right)V_{m}^{\left(t\right)}, \end{array}$$

with query, key, and value matrices

(7) $$Q_{m}^{\left(t\right)}=ZW_{Q,m}^{\left(t\right)},\;K_{m}^{\left(t\right)}=ZW_{K,m}^{\left(t\right)},\;V_{m}^{\left(t\right)}=ZW_{V,m}^{\left(t\right)},$$

where m=1,…,M indexes attention heads, $d_{m}=d/M$ is the head dimension, and $W_{Q,m}^{\left(t\right)},W_{K,m}^{\left(t\right)},W_{V,m}^{\left(t\right)},W_{O}^{\left(t\right)}$ are learnable projection matrices.

After *T* layers, we obtain the updated token matrix $Z^{\left(T\right)}$ and split it back into global and local parts:

(8) $$\left[\begin{array}{c} G^{\left(T\right)}\\ X^{\left(T\right)} \end{array}\right]=Z^{\left(T\right)}.$$

**Figure 4:**
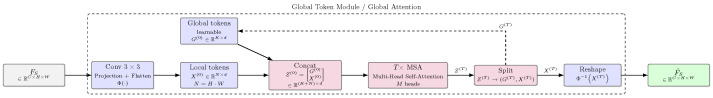
The detailed structure of the global token for the bottleneck module.

Finally, the refined local tokens are reshaped back to the bottleneck feature map

(9) $${\tilde{F}}_{S}=\Phi^{-1}\;\left(X^{\left(T\right)}\right)\in R^{C\times H\times W},$$

which is then fed into the decoder path of the U-Net. The resulting global-token module is shown in Figure 4.

### 3.2. Hybrid Adversarial–PSNR Loss

**Figure 5:**
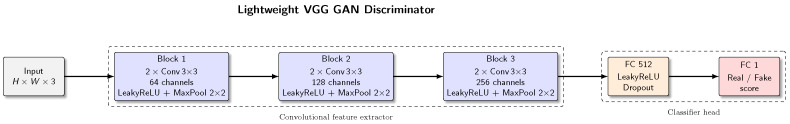
An additional discriminator outside the backbone network, mainly for generating adversarial losses.

The core of our method is to use the newly designed hybrid loss. To introduce adversarial loss, we use a lightweight VGG as the discriminator for the output of global-token U-Net, and its structure is shown in Figure 5. As such, global-token U-Net is a generator and its output also needs to be constrained by differences with the ground truth. Let *G* denote the generator and *D* the discriminator. Given a mini-batch of low-resolution inputs ${\left\{y_{i}\right\}}_{i=1}^{B}$ and corresponding high-resolution ground-truth images ${\left\{x_{i}\right\}}_{i=1}^{B}$, the generator produces ${\hat{x}}_{i}=G\left(y_{i}\right)$. We adopt a hinge-based adversarial loss combined with a PSNR-based regularization term.

**Adversarial loss.** This module was mainly inspired by Lucic et al. [20]. For the discriminator, the hinge adversarial loss is defined as

(10) $$L_{D}=\frac{1}{B}\sum_{i=1}^{B}\left[\max\left(0,\;1-D(x_{i})\right)+\max\left(0,\;1+D({\hat{x}}_{i})\right)\right].$$

The generator is trained to fool the discriminator via

(11) $$L_{\mathrm{adv}}^{G}=-\frac{1}{B}\sum_{i=1}^{B}D\left({\hat{x}}_{i}\right).$$

**PSNR-based regularization.** This module was mainly inspired by Chen et al. [19]. For each sample, we compute the mean-squared error (MSE) between ${\hat{x}}_{i}$ and $x_{i}$ as

(12) $${\mathrm{MSE}}_{i}=\frac{1}{P}∥{\hat{x}}_{i}-x_{i}{∥}_{2}^{2},\;P=3\cdot H_{\mathrm{out}}\cdot W_{\mathrm{out}},$$

where $H_{\mathrm{out}}$ and $W_{\mathrm{out}}$ denote the height and width, respectively. Given the maximum possible pixel value M=255 and a small constant $\varepsilon =1\times 10^{-4}$ for numerical stability, the per-sample PSNR is

(13) $${\mathrm{PSNR}}_{i}=10\log_{10}\left(\frac{M^{2}}{\max({\mathrm{MSE}}_{i},\varepsilon )}\right).$$

We define the PSNR-based loss as the negative batch-averaged PSNR:

(14) $$L_{\mathrm{PSNR}}=-\frac{1}{B}\sum_{i=1}^{B}{\mathrm{PSNR}}_{i}.$$

Minimizing $L_{\mathrm{PSNR}}$ is therefore equivalent to maximizing the PSNR of the reconstructed images.

**Generator objective.** The final generator loss is given by

(15) $$L_{G}=L_{\mathrm{adv}}^{G}+\lambda_{\mathrm{PSNR}}\;L_{\mathrm{PSNR}},$$

where $\lambda_{\mathrm{PSNR}}>0$ is a hyperparameter that refers to a weighting coefficient.

The adversarial term encourages perceptually realistic, high-frequency details, while the PSNR-based regularization explicitly penalizes deviations from the ground truth, thereby suppressing hallucinated structures and improving the trustworthiness of the reconstructed medical images.

## 4. Experiments

### 4.1. Dataset

Our experiment is based on the FIVES dataset [31]. This is a dataset with 800 fundus images taken by the same device. It is balanced across four categories: normal eyes, diabetic retinopathy, age-related macular degeneration, and glaucoma (200 images per category). All images are scanned in a consistent style at 2048×2048 resolution. To obtain the super-resolution dataset, we use the bicubic method to downsample the images:

(16) $$y_{i}={\mathrm{Down}}_{s}^{\mathrm{bicubic}}\left(x_{i}\right),$$

where ${\mathrm{Down}}_{s}^{\mathrm{bicubic}}\left(\cdot \right)$ denotes bicubic downsampling by a factor of *s*. The resulting pairs ${\left\{\left(y_{i},x_{i}\right)\right\}}_{i=1}^{N}$ constitute the supervised training set for medical image super-resolution. *s* is set to 8, thereby producing low-resolution images with a resolution of 256×256. Then we divide the training set and the test set in a ratio of 8:2.

### 4.2. Implementation Details

We implement our method using the deep learning framework PyTorch 2.5.1 [32] and the Adam [33] optimizer. The learning rate is set to $1\times 10^{-4}$ and gradually reduced to $1\times 10^{-5}$ using the cosine annealing method. The batch size is set to 8 and the model is trained using two Nvidia VGPUs (32 G, NVIDIA Corporation, Santa Clara, CA, USA) for a fixed 800 epochs. The hyperparameter $\lambda_{\mathrm{PSNR}}$ in the designed loss function is set to 0.1.

**Figure 6:**
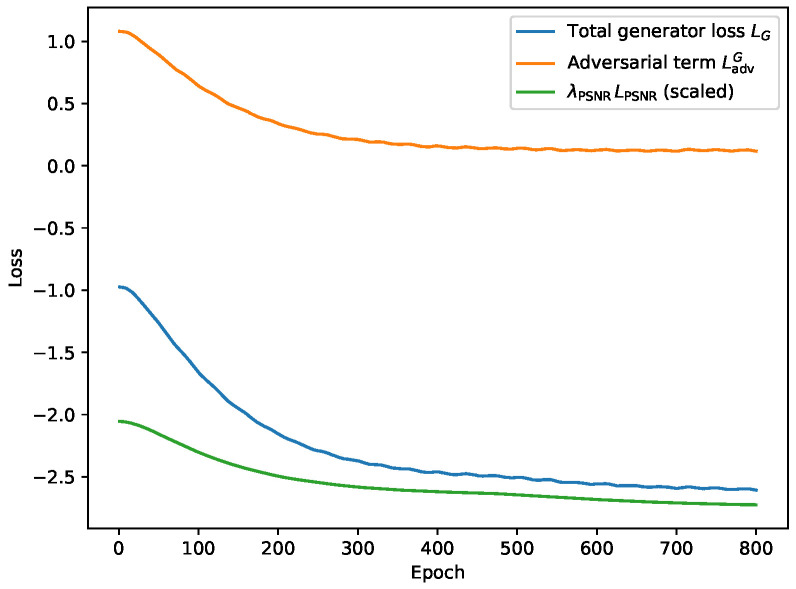
The training curve under the set implementation details.

The training curve of the hybrid loss during the training process is shown in Figure 6. As the training converges, the PSNR loss decreases more significantly, while the effect of the adversarial loss gradually decreases.

### 4.3. Metrics

We use SSIM to assess how similar the reconstructed results are to the ground truth in terms of structure and visual quality, and we use the PSNR to measure the pixel-level fidelity [34,35].

In addition to this, to evaluate the trustworthiness, we use LR-PSNR and LR-SSIM. These two metrics are calculated by downsampling the super-resolved image to the original resolution and calculating the related metrics with the original input image. Higher LR-PSNR and LR-SSIM indicate that the super-resolution results are faithful to the original details and have higher reliability.

In addition to the metrics for measuring fidelity to the original details, we have also introduced three general indicators of trustworthiness. LR-LPIPS is computed between $\mathrm{Down}(\hat{x})$ and y (lower is better). GradCons is the mean $L_{1}$ error of Sobel gradients, e.g., $∥\nabla \left(\mathrm{Down}\left(\hat{x}\right)\right)-\nabla \left(y\right){∥}_{1,\mathrm{mean}}$ (images normalized to [0,1]). HF-Err is the mean $L_{1}$ error of Laplacian/high-pass responses, e.g., $∥\Delta \left(\mathrm{Down}\left(\hat{x}\right)\right)-\Delta \left(y\right){∥}_{1,\mathrm{mean}}$ (lower is better).

### 4.4. Results

Table 2 compares the quantitative evaluation under the previous setting using indicators, including the general super-resolution methods and medical image super-resolution algorithms. The method we proposed demonstrates good reconstruction quality. Meanwhile, our method has reached a state-of-the-art level in LR-PSNR and LR-SSIM, demonstrating a higher degree of trustworthiness and reliability. Although PD-CR [7] and DiT4SR [36] based on diffusion models achieve higher PSNRs, their restoration of original details is still weaker.

**Figure 7:**
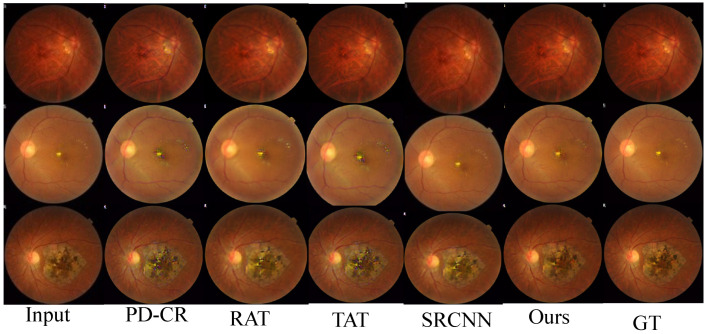
Visual effect comparison of super-resolution algorithms.

The intuitive qualitative comparison is shown in Figure 7, from which it can be seen that our method maintains the original details while achieving high visual quality.

### 4.5. Cross-Domain Evaluation

**Figure 8:**
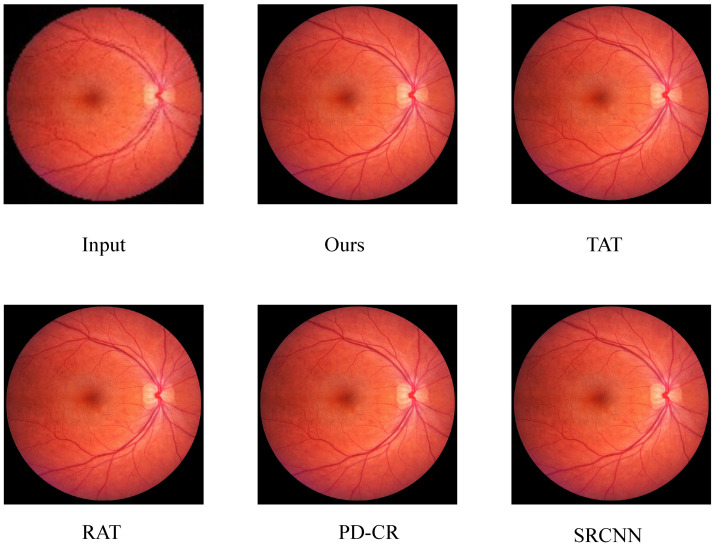
Cross-domain validation results without ground truth.

To demonstrate the generalization ability of the proposed method, we conducted cross-domain tests on the trained model using low-resolution fundus images without any ground-truth references. ODIR [38] is a multi-class fundus dataset. We performed super-resolution processing on the blurred downsampled images within this dataset and obtained ideal results, indicating that the proposed method can be applied to real super-resolution tasks on different devices, as shown in Figure 8.

### 4.6. Ablation Study

Our method obtains competitive LR-PSNR and LR-SSIM, fully ensuring the credibility of the original semantics of medical images. The source of trustworthiness lies in the combined influence of the hybrid loss function. To verify this, we conduct ablation experiments using a single loss function (PSNR or Adv.), and the quantitative results are shown in Table 3.

**Figure 9:**
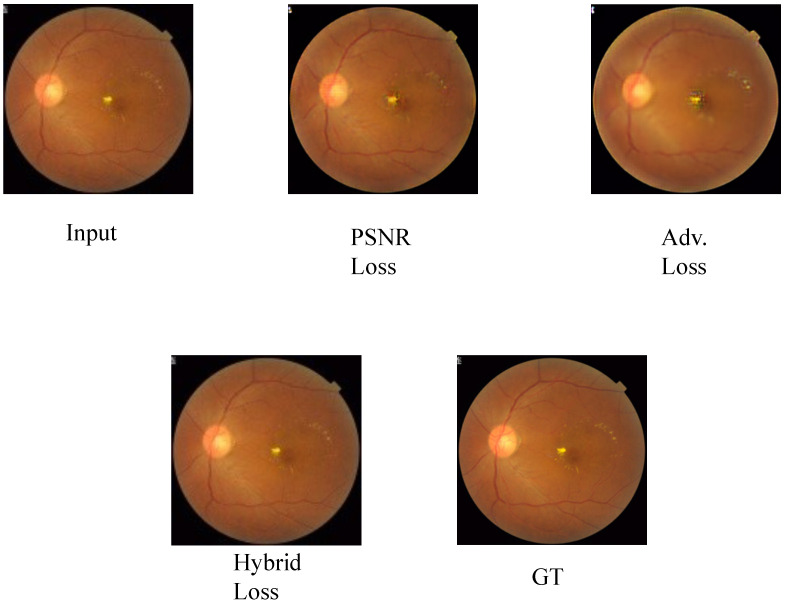
The result of the ablation experiment.

Combining the adversarial term with the PSNR-based regularization yields the best full-resolution PSNR/SSIM and restores LR-PSNR/LR-SSIM compared with the adversarial-only variant. This confirms that the proposed hybrid loss can effectively balance perceptual sharpness and input consistency, thereby enhancing the trustworthiness of medical image super-resolution. The qualitative visual quality in Figure 9 further illustrates this point.

The evaluations of different ablations are summarized in Table 4, indicating that the proposed method can take into account both visual quality and reliability.

### 4.7. Effect on High-Level Task

We give the practical auxiliary diagnosis application to demonstrate the effectiveness and credibility of the proposed method. The results show that the proposed method can effectively support the application of classification. The FIVES dataset [31] is naturally classified into four balanced categories based on different pathologies. We take all the super-resolution datasets formed after downsampling as the classification dataset. We employ a ResNet-50 network as the backbone and train it for 100 epochs for classification. Table 5 summarizes the accuracy comparison, indicating that our method can effectively assist in automatic classification diagnosis.

**Figure 10:**
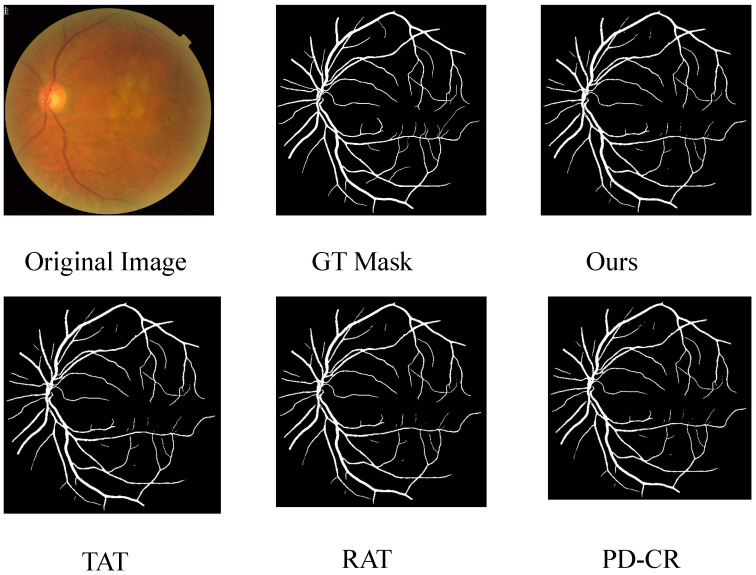
Vessel segmentation performance on FIVES using images reconstructed by different SR pipelines.

Segmentation is another important high-level task for clinical diagnosis. We construct a segmentation dataset using the super-resolved images generated by different methods, paired with the corresponding vessel masks provided in the FIVES dataset. The dataset is split into training and testing sets with a ratio of 0.8:0.2. A classical U-Net is trained for 100 epochs under the same settings for all methods. The quantitative and qualitative segmentation results are presented in Table 6 and Figure 10, respectively. The results demonstrate that the proposed method achieves better downstream performance, indicating stronger compatibility with high-level tasks and further supporting its credibility.

## 5. Conclusions

To address the trustworthiness issue of medical image super-resolution, we propose a comprehensive method. This method uses global-token U-Net as the backbone network, adds a lightweight VGG as the discriminator, and employs a hybrid PSNR and adversarial loss. Empirical results show that the proposed method can balance visual quality and reliability and ensure fidelity to the original details and is more suitable for auxiliary diagnosis. Future work can explore the role of other loss functions such as perceived loss.Some of the theoretical analyses of the methods presented in this paper still require exploration in future research. The validity of 3D imaging or other modalities still needs to be verified through more universal experiments.

## Figures and Tables

**Table 1:** Multi-aspect comparison of representative methods discussed in Section 2.1 and Section 2.2.

Sec.	Method	Paradigm	Input/Task	Degradation Setting	Halluc. Risk ^†^	Notes (Strengths vs. Trustworthiness Limitations)
2.1	GraphCutSR [21]	Optimization (graph-cut)	Multi-frame SR	Model-based; alignment-dependent	Low	Interpretable energy minimization and explicit regularization; typically faithful but limited representational power; sensitive to registration/motion errors and computationally heavier than feed-forward nets.
2.1	SRCNN [4]	CNN	Single-image SR	Usually synthetic (e.g., bicubic)	Low	Stable pixel-level fidelity as a baseline; limited capacity and tends to over-smooth; weak robustness to complex/unknown real degradations.
2.1	VDSR [5]	Deep CNN (residual)	Single-image SR	Usually synthetic (e.g., bicubic)	Low	Higher reconstruction accuracy via depth; still assumes a fixed degradation model; may sacrifice perceptual sharpness for fidelity under pixel-driven training.
2.1	EFATSR [22]	Transformer	Single-image SR	Synthetic degradations	Medium	Strong representation with long-range/frequency-aware modeling; performance and reliability can degrade under domain shift, where “plausible” high-frequency details may not be anatomically/physically grounded.
2.1	DFDAN [23]	CNN + attention	Single-image SR	Synthetic degradations	Medium	Efficient feature distillation and enlarged receptive fields; good efficiency/quality trade-off; trustworthiness largely depends on supervision realism and whether explicit consistency constraints are used.
2.1	ETCASR [8]	Transformer (efficient attention)	Stereo SR	Synthetic degradations + stereo cues	Medium	Exploits stereo correspondence for better detail recovery; requires stereo pairs and careful cross-view consistency to avoid introducing view-inconsistent “invented” structures.
2.1	DWTrans [1]	Diffusion + transformer	Single-image SR	Generative prior (learned)	High	Pretrained diffusion priors can boost perceptual realism; generative sampling increases hallucination risk unless strong data consistency and conservative objectives are enforced.
2.1	PD-CR [7]	Diffusion (constrained refinement)	Restoration/SR	Generative prior + refinement constraints	Medium–High	Constrained refinement can mitigate artifacts vs. unconstrained generative restoration; still slower and may hallucinate fine structures in ill-posed settings if constraints are insufficient.
2.2	SwinIR [18]	Transformer (Swin)	Restoration incl. SR	Mostly synthetic; can be fine-tuned	Medium	Strong universal backbone for SR/denoising/deblurring; not inherently medical-specific, so domain shift can yield anatomically implausible details without explicit fidelity constraints.
2.2	RAT [11]	Transformer (region attention)	Medical restoration incl. SR	Task-/modality-specific	Medium	Region-aware modeling can enhance salient structures; still requires explicit fidelity/anatomy constraints to suppress hallucinated content in ill-posed reconstructions.
2.2	HAT [27]	Transformer (hybrid attention)	SR backbone	Synthetic degradations	Medium	High-capacity SR transformer with strong detail synthesis; for medical usage, typically needs stronger input consistency regularization to avoid over-confident micro-textures.
2.2	SMambaUNet [25]	U-Net + Mamba	Medical SR	Modality-specific	Low–Medium	Combines U-shaped locality with long-range dependency modeling; “self-prior” can regularize outputs; trustworthiness remains data- and loss-dependent (especially under limited clinical data).
2.2	TAT [26]	Transformer (task-adaptive)	All-in-One MedIR	Multi-task/multi-degradation	Medium	Mitigates task interference and imbalance via task-adaptive weights and loss balancing; broader scope increases the need for conservative constraints to ensure anatomy-faithful SR across tasks/modalities.
2.2	InverseSR [10]	Latent diffusion	3D brain MRI SR	Generative prior (3D)	High	Diffusion priors can recover visually convincing 3D details; generative nature implies higher hallucination risk and typically demands strict data consistency and clinical validation.
2.2	CoSFNet [28]	Unified network (SR + motion)	4D MRI (motion+SR)	Motion + SR coupled model	Low–Medium	Joint motion estimation and SR improves temporal/spatial coherence; specialized pipeline and data requirements; explicit motion/consistency modeling can improve reliability.
2.2	MSFDecouple [29]	Multiscale fusion	Multimodal MRI SR	Cross-modal priors	Low–Medium	Leverages complementary modalities to stabilize SR; requires aligned multimodal inputs; potential bias if one modality is corrupted/misaligned (cross-modal “leakage”).

^†^ “Hallucination risk” is a qualitative indicator mainly driven by whether the method relies on explicit generative sampling (e.g., diffusion) versus feed-forward regression and whether strong input/data consistency mechanisms are typically present.

**Table 2:** Quantitative comparison on the FIVES test set for 8× super-resolution with additional trustworthiness metrics. PSNR/LR-PSNR are in dB. ↑ indicates higher values are better, ↓ indicates lower values are better. LR-* metrics (including LR-PSNR, LR-SSIM, LR-LPIPS) are computed between $\mathrm{Down}(\hat{x})$ and the original LR input y. DiT4SR and LMLT models are fine-tuned from their publicly available pre-trained weights.

Method	SR Quality vs. HR		Trustworthiness/LR Consistency vs. LR Input				
	PSNR ↑	SSIM ↑	LR-PSNR ↑	LR-SSIM ↑	LR-LPIPS ↓	GradCons ↓	HF-Err ↓
SRCNN [4]	25.8	0.83	49.6	0.99	0.010	0.006	0.006
VDSR [5]	26.4	0.84	49.3	0.98	0.014	0.008	0.008
RCAN [6]	27.0	0.86	49.1	0.98	0.016	0.009	0.009
PD-CR [7]	28.1	0.87	49.0	0.97	0.025	0.015	0.016
ETCASR [8]	27.2	0.87	49.1	0.99	0.012	0.007	0.007
RAT [11]	27.3	0.86	49.0	0.99	0.013	0.007	0.008
HAT [27]	27.4	0.87	49.2	0.99	0.011	0.006	0.007
DiT4SR [36]	28.3	0.88	48.9	0.97	0.028	0.017	0.018
LMLT [37]	27.3	0.87	49.3	0.99	0.010	0.006	0.006
Ours	27.6	0.88	49.9	0.99	0.006	0.004	0.0048

**Table 3:** Quantitative results of the ablation study on the FIVES test set (8× super-resolution). ↑ indicates higher values are better.

Method	PSNR ↑	SSIM ↑	LR-PSNR ↑	LR-SSIM ↑
PSNR Loss	27.4	0.87	49.02	0.99
Adv. Loss	27.3	0.88	49.12	0.99
hybrid loss	27.6	0.88	49.88	0.99

**Table 4:** Ablation study configuration: whether each component is enabled. ✓indicates that the corresponding method achieves the listed characteristic.

Method	High Visual Quality	High Evaluation Metrics	Trustworthiness
PSNR Loss		✓	
Adv. Loss	✓		
Our Method	✓	✓	✓

**Table 5:** Accuracy comparison. The outputs of all models are interpolated to 512×512 using bilinear interpolation as classification data. ↑ indicates higher values are better.

Input/Reconstruction Pipeline	Accuracy [%] ↑
GT	94.2
LR (×8)	86.5
Bicubic SR	88.3
SRCNN SR [4]	90.1
PD-CR SR [7]	91.3
RAT SR [11]	92.6
Ours (Global-token U-Net + hybrid)	93.0

**Table 6:** Vessel segmentation performance on FIVES using images reconstructed by different SR pipelines. All values are reported as mean ± std (sample size is omitted). Higher is better for IoU/Dice/Recall/Precision/Specificity, and lower is better for Hd/Hd95.

Method	IoU	Dice	Recall	Precision	Specificity	Hd	Hd95
InverseSR [10]	0.8437 ± 0.0910	0.9113 ± 0.0825	0.8908 ± 0.1150	0.9222 ± 0.1043	0.9951 ± 0.0017	45.4797 ± 27.8489	1.7270 ± 4.3492
TAT [26]	0.8502 ± 0.0645	0.9176 ± 0.0435	0.8911 ± 0.1150	0.9227 ± 0.1044	0.9951 ± 0.0017	45.3589 ± 26.8871	1.7248 ± 4.0370
SRCNN SR [4]	0.8515 ± 0.0645	0.9183 ± 0.0434	0.8917 ± 0.1151	0.9235 ± 0.1045	0.9952 ± 0.0017	44.4648 ± 26.4473	1.7536 ± 4.4060
PD-CR SR [7]	0.8515 ± 0.0644	0.9183 ± 0.0433	0.8918 ± 0.1151	0.9235 ± 0.1044	0.9952 ± 0.0017	43.4669 ± 26.1811	1.7403 ± 4.3984
Ours (Global-token U-Net + hybrid)	0.8524 ± 0.0644	0.9188 ± 0.0433	0.8922 ± 0.1151	0.9241 ± 0.1045	0.9952 ± 0.0017	43.4562 ± 26.0719	1.7483 ± 4.4092
GT	0.8543 ± 0.0645	0.9200 ± 0.0433	0.8931 ± 0.1152	0.9255 ± 0.1046	0.9953 ± 0.0017	42.2031 ± 23.8460	1.7845 ± 4.5530

## Data Availability

All relevant data are included in the article. Additional data are available from the corresponding author upon reasonable request.
